# Changes in Peptaibol Production of *Trichoderma* Species during In Vitro Antagonistic Interactions with Fungal Plant Pathogens

**DOI:** 10.3390/biom10050730

**Published:** 2020-05-07

**Authors:** Parisa Rahimi Tamandegani, Tamás Marik, Doustmorad Zafari, Dóra Balázs, Csaba Vágvölgyi, András Szekeres, László Kredics

**Affiliations:** 1 Department of Plant Protection, Bu Ali Sina University, Hamedan 6517833131, Iran; parisa_rahimi2003@yahoo.com (P.R.T.); zafari_d@basu.ac.ir (D.Z.); 2 Department of Microbiology, Faculty of Science and Informatics, University of Szeged, H-6726 Szeged, Hungary; mariktamas88@gmail.com (T.M.); dora.balazs91@gmail.com (D.B.); csaba@bio.u-szeged.hu (C.V.); andras.j.szekeres@gmail.com (A.S.)

**Keywords:** confrontation test, *Trichoderma*, phytopathogen, mycoparasitism, peptaibol

## Abstract

*Trichoderma* species are widely used as biofungicides for the control of fungal plant pathogens. Several studies have been performed to identify the main genes and compounds involved in *Trichoderma*–plant–microbial pathogen cross-talks. However, there is not much information about the exact mechanism of this profitable interaction. Peptaibols secreted mainly by *Trichoderma* species are linear, 5–20 amino acid residue long, non-ribosomally synthesized peptides rich in α-amino isobutyric acid, which seem to be effective in *Trichoderma*–plant pathogenic fungus interactions. In the present study, reversed phase (RP) high-performance liquid chromatography (HPLC) coupled with electrospray ionization (ESI) mass spectrometry (MS) was used to detect peptaibol profiles of *Trichoderma* strains during interactions with fungal plant pathogens. MS investigations of the crude extracts deriving from in vitro confrontations of *Trichoderma asperellum* and *T. longibrachiatum* with different plant pathogenic fungi (*Fusarium moniliforme*, *F. culmorum*, *F. graminearum*, *F. oxysporum* species complex, *Alternaria solani* and *Rhizoctonia solani*) were performed to get a better insight into the role of these non-ribosomal antimicrobial peptides. The results revealed an increase in the total amount of peptaibols produced during the interactions, as well as some differences in the peptaibol profiles between the confrontational and control tests. Detection of the expression level of the peptaibol synthetase *tex1* by qRT-PCR showed a significant increase in *T. asperellum/R. solani* interaction in comparison to the control. In conclusion, the interaction with plant pathogens highly influenced the peptaibol production of the examined *Trichoderma* strains.

## 1. Introduction

Various approaches are used to reduce the economic and aesthetic damages caused by plant pathogens, but most of them are not efficient enough. Due to microbial resistance to traditional antibiotics and chemical compounds, as well as the environmental and health risks of chemicals, scientists’ attempts are focused on finding safe and eco-friendly alternatives. In recent years, biological-based methods have been noticed to deal with these challenges [1]. The application of biofertilizers and biopesticides is a promising alternative for plant protection against the attack of different pathogens [2]. *Trichoderma* spp. are popular due to their visible direct effects on fungal pathogens and their capability to induce resistance in plants [3]. The genus *Trichoderma* was identified by Christiaan Hendrik Persoon in 1794, but its biocontrol ability as a mycoparasite on *Rhizoctonia* and *Sclerotinia* was described firstly by Weindling in 1934. Now, more than 300 *Trichoderma* species have been identified morphologically and genetically [4,5].

*Trichoderma* species (Hypocreales, Ascomycota) are widespread in different ecosystems [2]. During the past years, several studies have identified the mycoparasitic responses of *Trichoderma* species, including the direct attack and lysis of the plant pathogens [6]. *Trichoderma* species are powerful tools for the purposes of biological control due to the production and secretion of lytic cell-wall-degrading enzymes (CWDEs) [7], root colonization, nutrient competition, the induction of systemic resistance in plants [8] and the production of a broad spectrum of secondary metabolites such as peptaibols [9]. Investigation of gene expressions during mycoparasitic interactions with *Rhizoctonia solani* proved the upregulation of genes related to metabolism, especially the production of antibiotic metabolites [10].

Peptaibols are linear peptides with 5 to 20 amino acid residues produced by peptaibol synthetases, which are non-ribosomal peptide synthetase (NRPS) enzymes [11,12]. The presence of the unusual amino acids α-aminoisobutyric acid (Aib) and isovaline (Iva) means that the hydroxylated C-terminus and the N-terminal acetylated amino acids are unique characteristics of this group of antimicrobial peptides [11]. The structure of the NRPS enzymes is responsible for the amino acid order of the peptaibols [13]. The modules of the NRPSs are semiautonomous and capable of the activation, elongation and assembly of the amino acids. These modules contain adenylation (A), thiolation (T) and condensation (C) domains, which together represent one minimal repeating unit of the NRPSs [12,14]. The order of these units finally determines the primary structure of the peptaibols. The first reported *Trichoderma* NRPS was *tex1* described from *Trichoderma virens* [15]. Although *Trichoderma* species are known as the main peptaibol producers, peptaibols have also been reported from *Acremonium*, *Tylopilus*, *Boletus*, *Bionectria*, *Paecilomyces*, *Emericellopsis*, *Cephalosporium*, *Stilbella*, *Gliocladium* and *Tolypocladium* species [16].

Antimicrobial activities of peptaibols were reported against a number of fungi and Gram-positive bacteria, as well as viruses. Trichorzianines A and B [17], trichorzins HA and harzianines HC [18] from *T. harzianum*, trichokonins from *T. koningii* [19], tricholongins BI, BII [20] and longibrachins [21] from *T. longibrachiatum*, or trichotoxins from *T. asperellum* [22] are some examples showing the antimicrobial activity of *Trichoderma* peptaibols.

Trichotoxins are 18-residue peptaibols with trichotoxin-A-40s being firstly identified from *T. viride* NRRL5242 [23]. Later, trichotoxin-A-50s and other trichotoxin sequences were also described from *T. viride* [24] and *T. harzianum* [25], respectively. Brückner and Przybylski [23] classified the trichotoxins into the two subgroups of neutral (A-50) and acidic (A-40) trichotoxins separated based on their Gln17/Glu17 residue which defines the molecule to be neutral or acidic. Longibrachins are 20-residue peptaibols identified from *T. longibrachiatum* [26] which are also classified based on the Gln18/Glu18 exchange into neutral (longibrachin-A) and acidic (longibrachin-B) subgroups.

The mode of action of peptaibols is based on their physical, chemical and biological properties [11]. They are capable of forming voltage-dependent ion channels in plasma membranes [27], increasing membrane permeability [28] and consequently inducing cell death by cytoplasmic leakage [29]. The exogenous application of peptaibols enhances plant resistance against pathogens via triggering plant defence responses [30], e.g., trichokonins induced resistance in tobacco against tobacco mosaic virus (TMV) [31]. Moreover, on cucumber seedlings, the application of two synthetic 18-residue peptaibol isoforms (TvBI and TvBII) from *T. virens* Gv29-8 triggered responses of induced systemic resistance against leaf pathogenic bacteria, suggesting that 18-mer peptaibols act as inducers of non-cultivar-specific defence responses [32].

The synergistic effect between peptaibols and cell wall hydrolytic enzymes of *Trichoderma* during the antagonistic reaction against *Botrytis cinerea* has also been reported [33,34]. These data showed that peptaibols inhibit β-glucan synthase activity in the host fungus and act synergistically with β–glucanases; therefore, the destruction of the pathogen cell walls by *Trichoderma* species can be explained. In addition, peptaibols were considered as plant defense elicitors [35].

In this study, peptaibol profiles were analyzed during *Trichoderma*–fungal plant pathogen interactions in order to investigate the role of peptaibols in the antagonistic effect of *Trichoderma* species.

## 2. Materials and Methods

### 2.1. Fungal Strains and Culture Conditions

*T. asperellum* IRAN 3062C and *T. longibrachiatum* IRAN 3067C were derived from the Fungal Collection of Bu-Ali Sina University of Hamedan/Iran (BASUFC). To confirm the identity of the species, the translation elongation factor 1 alpha gene (*tef1*) was amplified and sequenced as described by Druzhinina et al. [36]. The amplified fragments contain partial sequence of the 4th large intron (first 207 nucleotides), the complete sequence of the 5th exon and partial sequence of the 5th short intron (last 71 nucleotides) of *tef1*. The 399 bp fragment of *T. asperellum* IRAN 3062C showed 100% sequence identity with *T. asperellum* strains GJS 06-294 [37] and NAIMCC-F-1763 [38], and the fragment of *T. longibrachiatum* IRAN 3067C with *T. longibrachiatum* strain CNM-CM 1798 [36].

*Fusarium moniliforme* SZMC 11046, *F. culmorum* SZMC 6239J, *F. graminearum* SZMC 6236J, *F. oxysporum* species complex (FOSC) SZMC 6237J, *Alternaria solani* SZMC 6241J and *Rhizoctonia solani* SZMC 21048 strains were derived from the Szeged Microbiology Collection (SZMC, www.szmc.hu). Different *Trichoderma*–plant pathogen combinations were used for direct dual interaction assays. All isolates were maintained on yeast extract-glucose agar media (10 g L^−1^ glucose, 10 g L^−1^ KH_2_PO_4_, 5 g L^−1^ yeast extract, 20 g L^−1^ agar).

### 2.2. Direct Confrontation Assays

**Figure 1:**
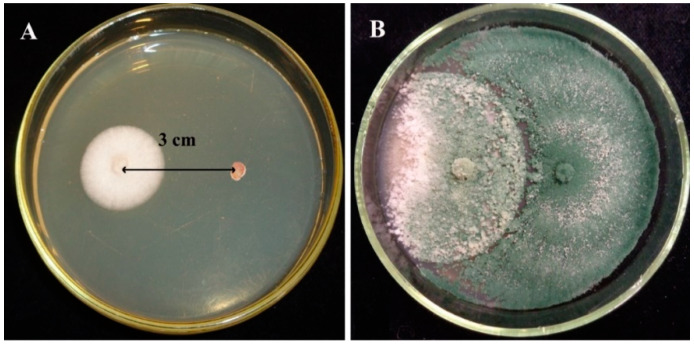
Direct confrontation test between *Trichoderma asperellum* IRAN 3062C and *Alternaria solani* SZMC 6241J after (**A**) 2 days and (**B**) 7 days on malt extract agar (MEA) medium.

In vitro antagonistic properties of the two *Trichoderma* strains were investigated based on the method described by Szekeres et al. [39]. Briefly, the experiments were performed in three parallel inoculations for *Trichoderma*–plant pathogenic fungus combinations. Plates containing plant pathogenic fungi or the *Trichoderma* strains (*T. asperellum* IRAN 3062C or *T. longibrachiatum* IRAN 3067C) alone were used as controls. Single agar plugs from the freshly growing mycelium of the particular plant pathogen were inoculated onto the surface of Petri plates (9 cm in diameter) containing malt extract agar medium (MEA) (5 g L^−1^ malt extract, 2.5 g L^−1^ yeast extract, 10 g L^−1^ glucose and 20 g L^−1^ agar) into a position 1.5 cm from the center of the plate. After 48 h in the case of the slower growing fungi like *R. solani*, *A. solani*, *F. moniliforme* and *F. graminearum*, or 24 h for the isolates with fast growing ability, such as *F. culmorum* and FOSC, a plug containing freshly grown mycelia of *T. asperellum* or *T. longibrachiatum* was inoculated in the same way, 3 cm apart from the plant pathogen’s inoculation position (Figure 1). The plates were kept at 25 °C for 16 h light and 8 h darkness per day. After 7 days the visible area of the *Trichoderma* colony and the total area occupied by both the colonies of *Trichoderma* and the plant pathogenic fungus were measured. Colony areas were used as a measurement tool to evaluate the antagonistic capacity of each isolate against the different plant pathogenic fungi using the free hand selection tool in Image J (https://imagej.nih.gov/ij/). Biocontrol index (BCI) values (area of the *Trichoderma* colony divided by the total area occupied by both *Trichodema* and the plant pathogen × 100) were used for calculating the antagonistic ability of each strain.

### 2.3. Peptaibol Extraction

Solvents used for extraction were methanol and chloroform (VWR International, Debrecen, Hungary). The surface of each plate (*Trichoderma* colony as control, plant pathogen colonies as controls, and *Trichoderma* plus plant pathogen in confrontational tests) was flooded twice with 5 mL chloroform, washed several times, collected in clean test tubes and shaken at room temperature for 2 h. After that, chloroform was evaporated to dryness by a rotary evaporator (IKA RV 10; IKA Works, Wilmington, NC, USA). The crude extracts were dissolved in methanol and collected in new tubes. *Trichoderma* spores and mycelia were pelleted by centrifuging at 10,000 rpm for 10 min (Biofuge Primo centrifuge, Heraeus, Hanau, Germany). The supernatant was transferred into new tubes and evaporated under nitrogen. The remaining dry material was dissolved in 200 µL methanol and kept at −20 °C for further analysis [40].

### 2.4. Biological Activity of the Crude Extracts

Approximate total amounts of peptaibols produced by each *Trichoderma* isolate were estimated by the rapid pre-screening method described by Marik et al. [41]. Antimicrobial activities of the crude extracts from strains *T. asperellum* IRAN 3062C and *T. longibrachiatum* IRAN 3067C were evaluated against *Micrococcus luteus* strain SZMC 0264. The inhibition zone was measured after 2 days and the total amount of peptaibols was estimated according to the calibration curve of alamethicin standard (Sigma-Aldrich, Budapest, Hungary) [41].

To evaluate antifungal activity of the crude extract, 1 mL of spore suspension (5 × 10^5^ spore per mL) of *F. moniliforme*, *F. culmorum*, *F. graminearum*, FOSC, *A. solani* and *R. solani* strains was spread on the surface of the plate containing MEA and 5 mm diameter wells were bored into the agar circularly. Twenty µL of the series dilution of the crude extract (200, 100, 50 and 10 µg mL^−1^ crude peptaibol extracts) was poured into the well and methanol was used as negative control. The plates were incubated at 25 °C for one week. Three parallel experiments were set up to measure the inhibition zones [33].

### 2.5. Identification and Semi-Quantitation of Peptaibols

The measurements were carried out with an Agilent 1100 modular reversed phase (RP) high-performance liquid chromatography (HPLC) system (Agilent, Palo Alto, CA, USA) coupled to a Varian 500 (Agilent, Palo Alto, CA, USA) ion trap mass spectrometer equipped with electrospray ion source (ESI-MS). Chemstation B.02.01 and MS Workstation 6.6 softwares controlled the HPLC and the MS, respectively. The extracts were separated and their full scan and MS^2^ spectra were acquired, and microheterogenous mixtures of peptaibols were sequenced using consecutive fragmentation in ESI-MS^n^ according to the method described by Marik et al. [42]. The total peptaibol production of each measurement was calculated based on the summarized integrated area of the sodiated molecular ions [M + Na]^+^ proportionated with their respective BCI values. For the identified peptaibols, the previously defined nomenclature was applied, where the relative amount of each peptaibol was expressed in percentages based on the peak area of the [M + Na]^+^ appearing in the full scan measurements [42].

### 2.6. RNA Extraction and qRT-PCR for Detection of tex1 Gene Expression

Total RNA was extracted by the method described by Orek [43] from *T. asperellum* (both as control and in confrontation with *R. solani*) with some modifications. RNA quality and quantity were checked by Nano drop (NanoPhotometer^®^ N120, IMPLEN, München, Germany), considering the ideal absorbance ratio (1.8 ≤ A260/280 ≤ 2.0). Total extracted RNA was also electrophoresed on 1% agarose gel to visualize the RNA integrity. For cDNA synthesis, approximately 5 mg of RNA was treated with DNase I, RNase-free (Pub. No. MAN0012000, Thermo Fisher Scientific Inc., Waltham, MA, USA) according to the manufacturer’s instructions. cDNA was synthesized using High-Capacity cDNA Reverse Transcription kit and Oligo dT primers (Yekta Tajhiz Azma, Tehran, Iran, Cat No. YT4500) according to the manufacturer’s manual. Real-time quantitative PCR was performed with gene-specific primers for *tex1*: TEX1F 5’-TTTCAGCGACGTTTTGCCAG-3’ and TEX1R 5’-TGGTGCAAAAATCGCACAGG-3’ amplifying a 186 bp fragment of the *tex1* NRPS gene were designed based on the sequence of *T. virens* Gv29-8 non-ribosomal peptide synthetase (*tex1*) (NCBI Reference Sequence: XM_014097635.1). qPCR reactions were carried out with 48 wells StepOne Real-Time PCR System (Applied Biosystems StepOne, Foster City, CA, USA), using SYBR^®^ Green. Each reaction was performed in 20 µL containing 1× Power SYBR Green PCR Master Mix (Yekta Tajhiz Azma, Tehran, Iran, Cat No. YT2552), 120 nM of primers (Pishgaman Co., Tehran, Iran) and 2 µL diluted (1/20) cDNA sample. PCR was performed using the thermal cycling parameters as follows: 95 °C, 10 min; and 40 cycles of 95 °C, 15 s; 58 °C, 25 s and 72 °C, 35 s. The qPCR data were analyzed by the 2^−ΔΔCT^ method [44]. The β-tubulin gene was used to determine the relative expression level of the *tex1* gene analyzed in this work. For the determination of qPCR efficiency of each primer pair, a standard curve was performed using cDNA dilutions of 1:5, 1:25, 1:125 and 1:625. Each sample was examined in duplicate. The corresponding qPCR efficiencies (E) were calculated for each primer pair with the LinRegPCR software [45]. In this way, the Real-time PCR Data Markup Language (RDML) file of fluorescence change data by StepOne software was analyzed by LinRegPCR software and qPCR efficiencies (E) were calculated. The efficiency obtained in both methods was approximately similar.

### 2.7. Statistical Analysis

In order to determine the significant differences between the peptaibol production of *Trichoderma* isolates in the confrontation assays with plant pathogens in comparison to the respective controls, peptaibol percentage level (the graph area of specific peptaibol compound divided by the area of the total peptaibol production) changes were calculated by each peptaibol peak area with the MS Data Review software. All data were analyzed for statistical significance by Graphpad Prism software version 6.01 (GraphPad Software, San Diego, CA, USA; www.graphpad.com). Comparative studies of different isolates were done by multiple *t* test analysis. The statistical significance was determined using the Holm–Šidak method with alpha = 5.00%. The statistical analysis of qPCR results was performed in SAS 9.4 (www.sas.com) in a completely randomized design with one-way ANOVA and Duncan’s test (*p* < 0.05).

## 3. Results

### 3.1. Inhibitory Activity of Peptaibol Extracts

**Figure 2:**
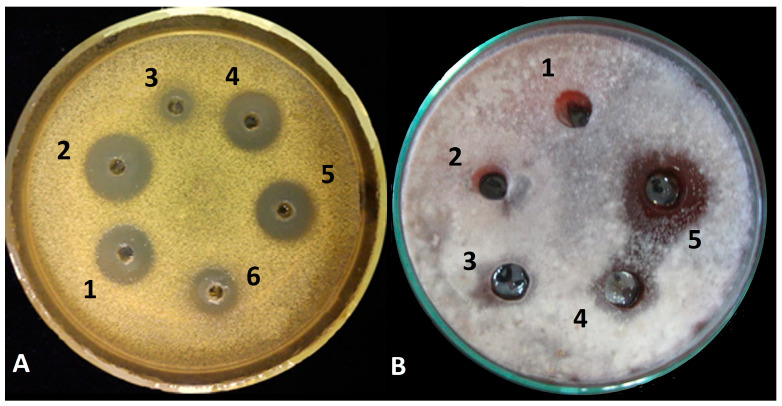
Biological activity assay of crude extracts from *Trichoderma* species against (**A**) *Micrococcus luteus*; 1, 2, 4 and 5: *Trichoderma asperellum* IRAN 3062C (604.28 µg mL^−1^), 3 and 6: *Trichoderma longibrachiatum* IRAN 3067C (230.1 µg mL^−1^) and (**B**) *Alternaria solani*; 1: methanol as control, 2: 10, 3: 50, 4: 100, 5: 200 µg mL^−1^ of peptaibol extract from *T. asperellum* IRAN 3062C.

The crude extracts of *Trichoderma* isolates used in this study showed antibacterial activities towards *M. luteus* (Figure 2A). *T. asperellum* IRAN 3062C had a larger inhibition zone (22 mm in diameter) than *T. longibrachiatum* IRAN 3067C. The alamethicin equivalent concentrations of the average amount of peptaibols in the crude extracts were 604.28 and 230.1 µg mL^−1^ for *T. asperellum* IRAN 3062C and *T. longibrachiatum* IRAN 3067C, respectively.

According to the results of the antifungal assay, the crude extract of *T. asperellum* inhibited spore germination and its effect started from the concentration of 100 µg mL^−1^ for all plant pathogenic fungi. The largest inhibition zones were seen in the case of *A. solani* (Figure 2B), *R. solani* and *F. moniliforme*. The results were in conformity with the findings of the BCI index assay described below.

### 3.2. Confrontation Experiments

**Figure 3:**
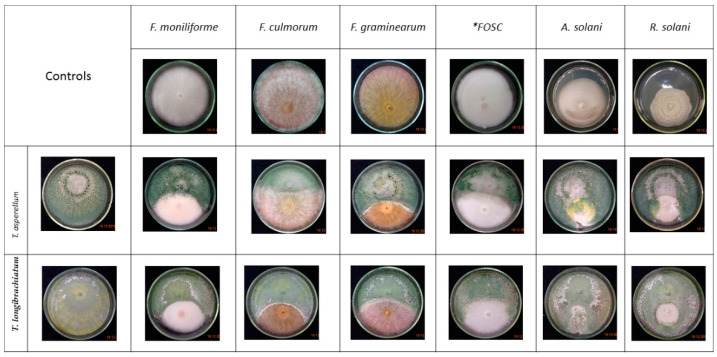
In vitro confrontation experiments between *Trichoderma asperellum* IRAN 3062C, *Trichoderma longibrachiatum* IRAN 3067C and plant pathogenic fungi on MEA after 7 days. * FOSC: *Fusarium oxysporum* species complex.

The dual culture technique (Figure 1) revealed that the examined *Trichoderma* strains were capable of overgrowing the examined plant pathogenic fungi. The mycelial growth of the plant pathogens was mitigated and a reduction of the colony diameter extension appeared in the presence of *Trichoderma* isolates (Figure 3).

**Figure 4:**
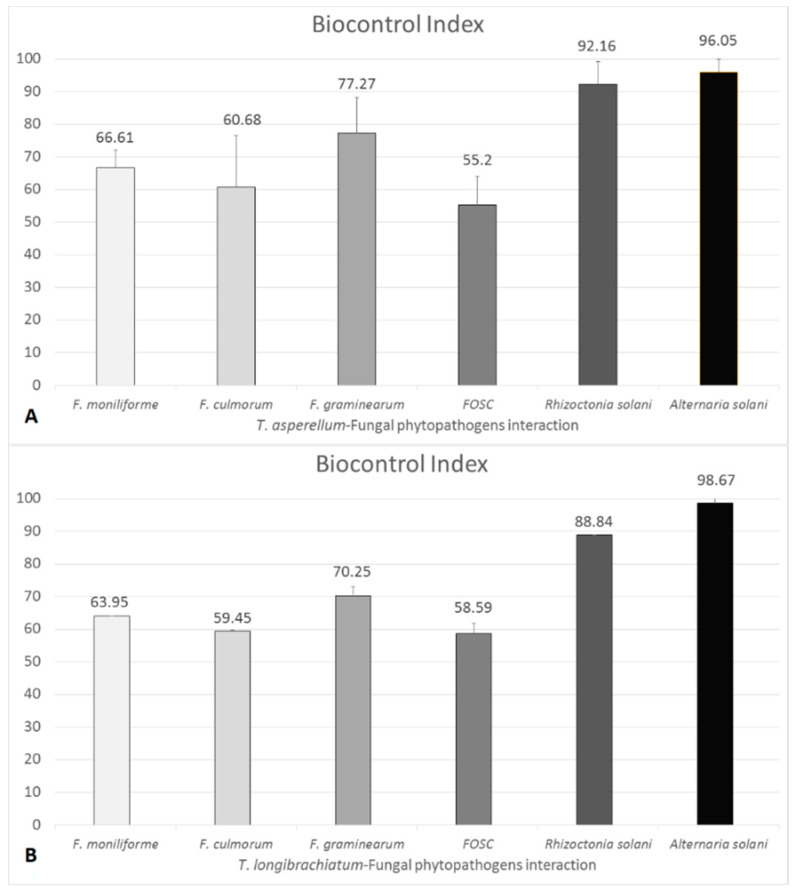
Biocontrol index (BCI) values of *Trichoderma asperellum* IRAN 3062C (**A**) and *Trichoderma longibrachiatum* IRAN 3067C (**B**) towards different plant pathogenic fungi. Bars represent standard deviation.

BCI values showed diversity for each *Trichoderma*–plant pathogen interaction according to the plant pathogenic fungus and could reach up to 100%. BCI values showed that *T. asperellum* IRAN 3062C has efficient antagonistic abilities against *A. solani* and *R. solani* with BCI values of 96.05% ± 3.8% and 92.16% ± 6.94%, respectively. The *T. longibrachiatum* strain IRAN 3067C used in this study showed lower antagonistic capacities than *T. asperellum* IRAN 3062C against the various plant pathogens. The interactions of *T. asperellum* and *T. longibrachiatum* producing trichotoxins and longibrachins, respectively, revealed BCI values in decreasing order against *A. solani*, *R. solani*, *F. graminearum*, *F. moniliforme*, *F. culmorum* and FOSC (Figure 4).

### 3.3. Changes in Peptaibol Production during Different In Vitro Trichoderma–Plant Pathogen Interactions

**Figure 5:**
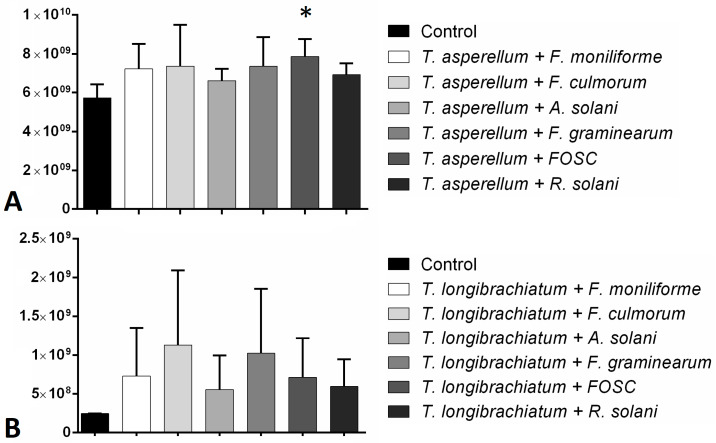
Total amounts of peptaibols produced by *Trichoderma asperellum* IRAN 3062C (**A**) and *T. longibrachiatum* IRAN 3067C (**B**) in interaction with plant pathogenic fungi (*Fusarium moniliforme*, *F. culmorum*, *F. graminearum*, FOSC, *Alternaria solani*, *Rhizoctonia solani*) compared with the control (*Trichoderma* without plant pathogen) in confrontation tests. Asterisks indicate significant differences, as determined by Student’s *t* test (*p* < 0.05) corrected with the Holm–Šidak method.

The total amount of peptaibols proved to be elevated during the interaction of *T. asperellum* with the tested plant pathogens and the increase proved to be significant in the case of the interaction with FOSC (Figure 5). The peptaibol production of *T. longibrachiatum*–plant pathogen interactions was also increased, although significant differences could not be observed.

**Figure 6:**
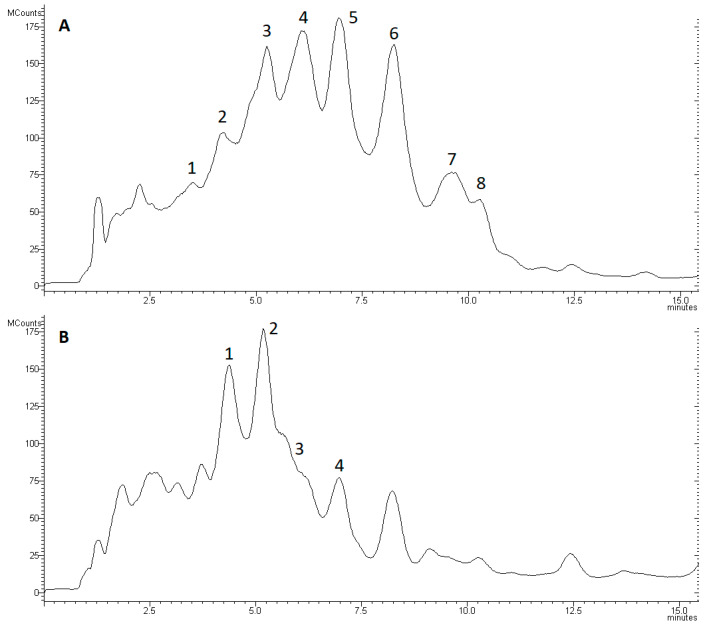
Total ion chromatogram of peptaibols extracted from (**A**) *Trichoderma asperellum* IRAN 3062C and (**B**) *Trichoderma longibrachiatum* IRAN 3067C. Kromasil C18; 5 µm, 1.6 × 250 mm; MeOH/H_2_O (86:14), flow rate 1 mL min^−1^. Numbers indicate peaks containing peptaibols (see Table 1).

Eight main peaks including the trichotoxin peptaibol group [22] as complex mixture of isoforms (Table 1) were detected in the chromatograms of *T. asperellum* IRAN 3062C (Figure 6A).

**Figure 7:**
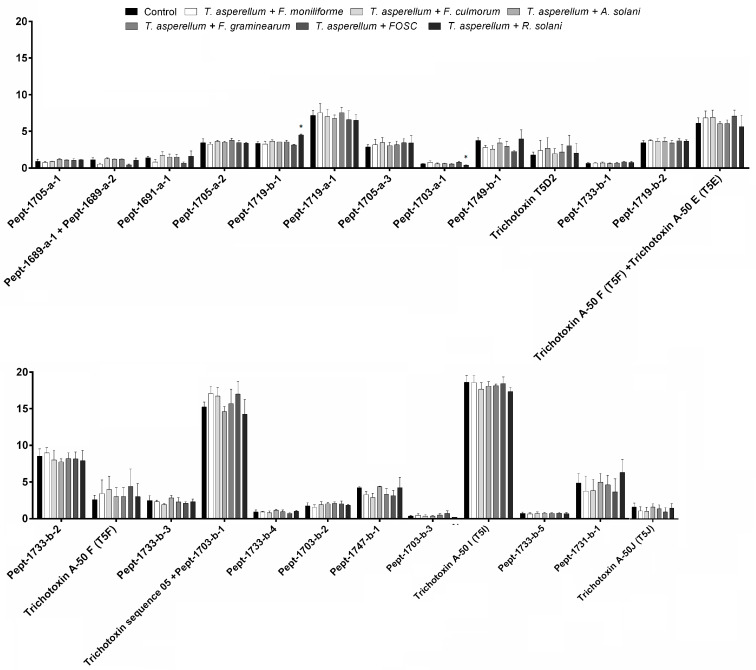
Relative amounts of peptaibols by *Trichoderma asperellum* IRAN 3062C in interaction with plant pathogenic fungi (*Fusarium moniliforme*, *F. culmorum*, *F. graminearum*, FOSC, *Alternaria solani*, *Rhizoctonia solani*) compared with the control (*Trichoderma* without plant pathogen) in confrontation tests. Asterisks indicate significant differences, as determined by Student’s *t* test (* *p* < 0.05) corrected with the Holm–Šidak method.

Analysing the mass spectrum of peak 1 revealed the presence of Pept-1705-a-1 [46] (Figure 6A). In peak 2, the peptaibols named Pept-1689-a-1 and a-2 [46] were different from the previously reported trichotoxins such as T5F [23] in the Aib residue at position 10. These two compounds were shown on the chromatograms (extracted to their [M + Na]^+^ ions) in one merged peak and their relative amounts have not significantly changed in any of the interactions. This peak also contains Pept-1691-a-1. In peak 3, signals related to five peptaibols, Pept-1719-a-1, Pept-1719-b-1, Pept-1705-a-3, Pept-1703-a-1 and Pept-17049-b-1 [46] were detected. These peptaibols were different from other detected trichotoxins with the presence of Ser in position 10. Pept-1705-a-2 proved to be different from Pept-1705-a-1 in Aib-Ala residues in positions 9 and 11 instead of Ala-Aib [25]. The production of Pept-1719-b-1 increased, while the amount of Pept-1703-a-1 decreased significantly (*p* < 0.05) in the *T. asperellum*–*R. solani* interaction. The analysis of peak 4 enabled the identification of Pept-1719-b-2, Pept-1733-b-1 and -b-2 [46], as well as trichotoxin T5D2, trichotoxin A50 E and F (Pept-1689-b-1 and -a-3) [25]. On the [M + Na]^+^ extracted chromatograms of trichotoxin A50 E and F, the peptides were also merged in one peak similarly to Pept-1689-a-1 and a-2 mentioned above. In peak 5, the signals related to trichotoxin A-50 F (Pept-1689-b-2), trichotoxin sequence 05 (Pept-1703-a-2) [25], Pept-1733-b-3, Pept-1703-b-1 and Pept-33-b-4 [46] were detected. Trichotoxin sequence 05 and Pept-1703-b-1 were also seen together in the same extracted [M + Na]^+^ peaks. In peak 6, five peptaibols were observed: Pept-1703-b-2, Pept-1747-b-1, Pept-1703-b-3, Pept-1733-b-5 [46] and trichotoxin A-50I (Pept-1717-b-1) [25]. Pept-1731-b-1 was found in peak 7, while peak 8 contained trichotoxin A50J (Pept-1731-b-2) [46] (Figure 7). No significant differences (*p* < 0.05) could be detected in the relative amounts of peptaibols in peaks 4–8 in any of the interactions.

**Figure 8:**
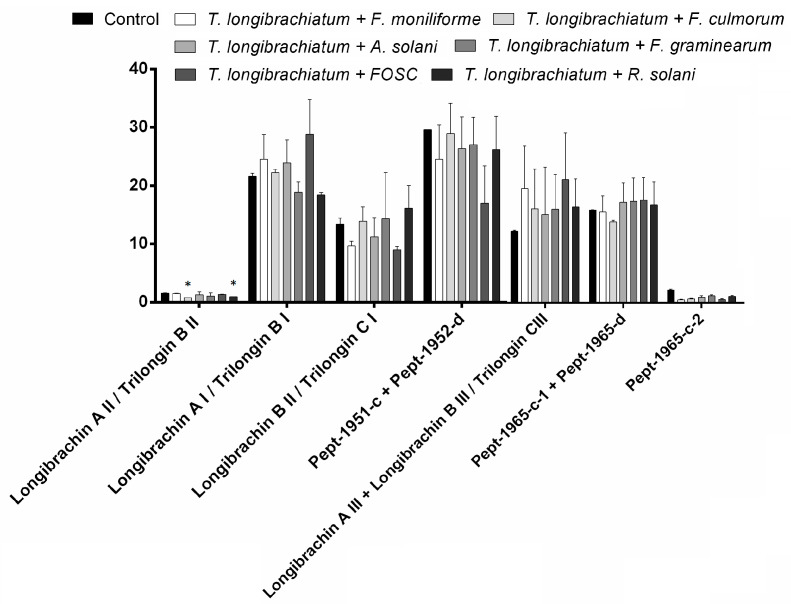
Relative amounts of peptaibols identified from *Trichoderma longibrachiatum* IRAN 3067C in interaction with plant pathogens compared with the control (*Trichoderma* without plant pathogens) in confrontation tests. Asterisks indicate significant differences, as determined by Student’s *t* test (* *p* < 0.05) corrected with the Holm–Šidak method.

As *T. longibrachiatum* is clinically important, it is not suggested to be used as a biocontrol agent [47]. The *T. longibrachiatum* strain was involved in this study as a producer of 20-residue long peptaibols (Figure 8). Longibrachins AI and AII were found in peak 1, while AIII was observed in peak 2 [48]. The relative amount of longibrachin AII was significantly decreased in interactions with *F. culmorum* and *R. solani*. Longibrachin BII and BIII could be identified in peaks 1 and 2, respectively [26,49]. Pept-1951-c and Pept-1952-d [46] could also be observed in the same merged extracted [M + Na]^+^ ion peak, similarly to longibrachin AIII and BIII, and Pept-1965-c-1 and Pept-1965-d. Pept-1966-d [46] and Pept-1965-c-1 [46] were found in peak 3 while Pept-1965-c-2 [46] could be observed in peak 4 (Figure 8).

### 3.4. Expression of the tex1 Gene during T. asperellum–R. solani Interaction

**Figure 9:**
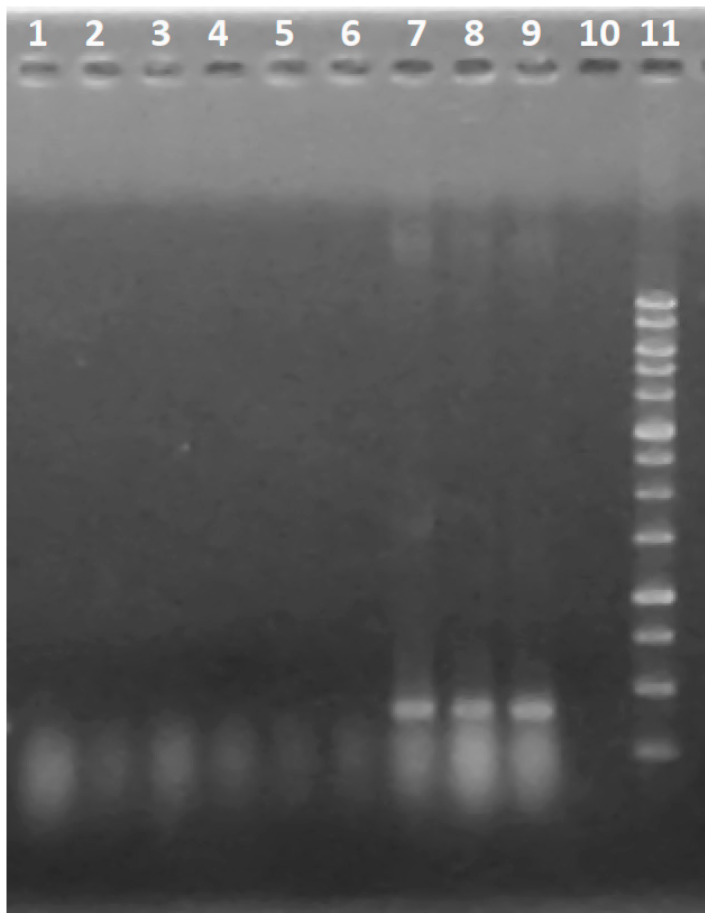
Amplification of *tex1* by PCR from *Fusarium moniliforme* SZMC 11046 (1), 2. *Fusarium culmorum* SZMC 6239J (2), *Fusarium graminearum* SZMC 6236J (3), FOSC SZMC 6237J (4), *Alternaria solani* SZMC 6241J (5), *Rhizoctonia solani* SZMC 21048 (6) *Trichoderma asperellum* IRAN 3062C (7) *T. asperellum* BS3-8 [50] (8), *Trichoderma longibrachiatum* IRAN 3067C (9), and H_2_O as negative control (10). Molecular weight marker: 100 bp ladder (11).

**Figure 10:**
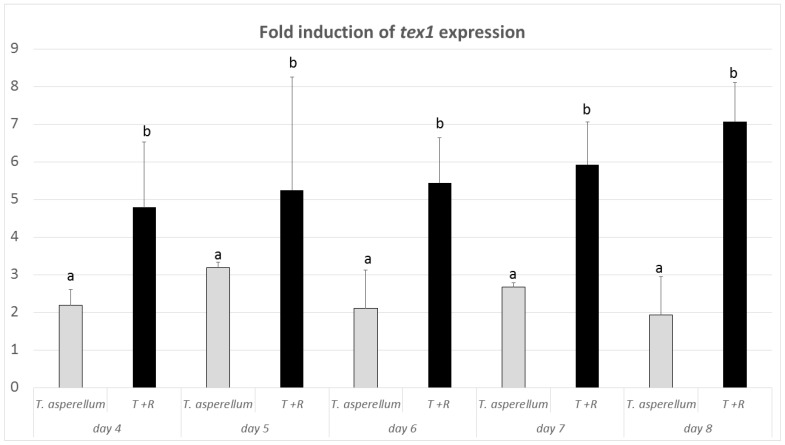
Quantitative RT-PCR analysis of *tex1*. Values (2^−ΔΔ*CT*^) correspond to relative measurements against the *tex1* transcript in *Trichoderma asperellum* IRAN 3062C (T) and *T. asperellum* IRAN 3062C challenged with *Rhizoctonia solani* (T + R). β-tubulin was used as an internal reference gene. Bars represent standard deviations of the mean values of three biological replicates. Different letters (a and b) indicate significantly different values (*p* < 0.05).

The results of DNA amplification from *T. asperellum* IRAN 3062C using the designed primers showed that the desired fragment (186 bp) of the *tex1* peptaibol synthetase gene could be amplified, while it was absent in the tested plant pathogenic fungi (Figure 9). The qRT PCR revealed that the mRNA level of *tex1* had been increased in *T. asperellum* IRAN 3062C in contact with *R. solani* in comparison to the control (*T. asperellum* IRAN 3062C alone) (Figure 10).

## 4. Discussion

*Trichoderma* species are prolific producers of secondary metabolites including peptaibols, epipolythiodioxopiperazines (ETPs), volatile and nonvolatile terpenes, pyrones, polyketides, and siderophores [12]. A genomic comparison performed between both saprophytic and parasitic species of *Trichoderma* revealed that the presence of some special compounds may be involved in rhizosphere competence and interactions of *Trichoderma* with plants [51] or in the killing of other microorganisms, and may also affect the plant–pathogen interactions and mycoparasitism [52].

Peptaibols are dominant secondary metabolites of filamentous fungi, including the ones exhibiting a mycoparasitic lifestyle, especially *Trichoderma* species [53,54]. They play an important role in the mycoparasitic interactions as well as in induction of induced systemic resistance (ISR) in plants via upregulation of the jasmonic acid and salicylic acid synthesis [30,32]. This study was conducted to survey the changes in the peptaibol production during mycoparasitic interactions.

Even though the BCI values are not able to precisely reflect the antagonistic abilities of a particular strain, using the dual culture confrontation tests in order to get analysable data for comparison of fungal biocontrol isolates is very popular [55]. By this method, the antagonistic potential of each *Trichoderma* isolate can be evaluated, as the most important mechanisms of antagonism, especially competition for space and nutrients, production of antifungal metabolites and mycoparasitism may all potentially occur on the confrontation plates [39]. Most of the *Trichoderma* strains involved in this study attacked the examined plant pathogenic fungi and overgrew their mycelia. In other cases, the observation of an inhibition zone between the two fungi indicated the production of certain antifungal metabolites by the *Trichoderma* isolates. In all the interaction tests, the growth rate of the plant pathogen was reduced in contact with *Trichoderma*. Strain *T. asperellum* IRAN 3062C had the highest BCI values against *R. solani* and *A. solani*, thus it can be considered as a promising biocontrol agent (BCA) against these plant pathogens. Among the four taxa of *Fusarium* tested in this study, the highest BCI values were determined for *T. asperellum* IRAN 3062C against *F. graminearum*. Regarding the results against the two other taxa, *F. culmorum* and FOSC, which are both soil-borne plant pathogenic fungi, it seems that *T. asperellum* had lower BCI values in interaction with soil-borne *Fusarium* species.

The antibiotic activity of a peptaibol designated as U-21963 from *T. viride* (later reidentified as *T. arundinaceum* [56]) against several species of fungi and bacteria was firstly reported in 1966 by Meyer and Reusser [57]. Peptaibols are mostly effective against a range of Gram-positive bacteria and fungal phytopathogens [58]. Previous work on the diversity profiles of peptaibols produced by *T. aggressivum* and *T. pleuroti* in interactions with *Agaricus bisporus* and *Pleurotus ostreatus* showed that peptaibols potentially inhibit the growth of mushroom mycelia. Furthermore, significant changes could be observed in the peptaibol profile of the *Trichoderma* strains during the interaction with their respective hosts [59]. In the present study, analysing the effect of crude peptaibol extract on spore germination showed that 200 µg mL^−1^ of total peptaibol concentration inhibited the spore germination of all tested phytopathogenic fungi. Regarding the role of cAMP-dependent protein kinase polarity and signal transduction in spore germination and formation of germ tube [60], it is necessary to focus on the effect of peptaibols in these processes. A study performed on trichorzin HA V (Ac-Aib-Gly-Ala-Aib-Iva-Gln-Aib-Val-Aib-Gly-Leu-Aib-Pro-Leu-Aib-Iva-Gln-Leuol) and alamethicin (Ac-Aib-Pro-Aib-Ala-Aib-Ala-Gln-Aib-Val-Aib-Gly-Leu-Aib-Pro-Val-Aib-Aib-Glu-Gln- Pheol) revealed that trichorzin HA V elevates cAMP levels and selectively interacts with the calcitonin receptor [61], while alamethicin does not. Since cAMP signaling is necessary for many cellular physiological processes such as spore germination in *Schizosaccharomyces pombe* [62], as well as growth, germination, mycoparasitism and secondary metabolism in *Trichoderma* [63], it seems that even a single amino acid difference in a sequence may completely change the biological activity of a peptaibol.

It was shown in another study [46] that the 18-residue peptaibols produced by *T. asperellum* IRAN 3062C generally belong to the A50 group of trichotoxins [22]. Trichotoxins have a unique general structure with Gly followed by Aib in positions 2 and 3 [53]. Trichotoxins differ from each other in Ala/Aib in positions 9 and 11 (Table 1). This group of peptaibols was firstly isolated in 1984 [64] and distinguished from peptaibols such as alamethicin by the different primary structure. This group of peptaibols shorter than alamethicin has been isolated from different species of *Trichoderma* in acidic or neutral forms based on the presence of Glu/Gln at the end of the sequence in position 17 [23]. The C-terminal alcohol residue of these groups of peptaibols is valinol. Acidic (TT-A40) and neutral (TT-A50) trichotoxins have different polarization [24]. All the isolated peptaibols detected in this study belong to the neutral trichotoxins. Trichotoxins belong to the sub-family 1 of peptaibols. The high number of Aib residues in the sequence is evidence of α-helical structure [65]. It was noted in the literature that one Gln residue near the middle of the sequence (frequently in position 6 or 7) and another one in the C-terminal play a conducting role in the pore lumen [66]. Another amino acid proposed to be involved in the mechanism of insertion into membrane bilayers is Pro in position 13 [29]. In the present study, Pept-1703-b-2 was the only detected trichotoxin with Gly in position 11 (Table 1). Helix bending, flexibility and insertion are the functions proposed for this residue [29]. However, based on our data the amount of this peptaibol did not show any considerable changes during the interactions, furthermore, it was produced in very small quantities.

Analysis of the MS chromatograms of crude extracts from control and confronted cultures revealed that *T. longibrachiatum* IRAN 3067C produced 20-residue peptaibols belonging to the longibrachin group from subfamily 1 of peptaibols [46]. These long peptaibols were firstly described by Leclerc et al. [21] from a liquid culture medium of *T. longibrachiatum* and have a high proportion of Aib, therefore conforming to an α-helical structure [67]. The high mean hydrophobicity of the primary structure gives them the ability to form channels in membrane bilayers [17]. The Glu residue in the C-terminus and the Pro-2 residue in the N-terminus of the peptide helix have the major role in bilayer interface [68]. All the isolated peptaibols from *T. longibrachiatum* have the amino acid Gly in position 11. This is the same position two steps before the unstable Aib–Pro bond as in Pept-1703-b-2 produced by *T. asperellum* IRAN 3062C, which also has Gly at this position and is unique among the peptaibols produced by this strain. Out of the 10 identified peptaibols of *T. longibrachiatum* IRAN 3067C, the 5 longibrachins have the general formula of [Ac-Aib-Ala-Aib-Ala-Aib-(Aib/Ala)-Gln-Aib-Vxx-Aib-Gly-Lxx-Aib-Pro-Vxx-Aib-(Aib/Vxx)-(Gln/Glu)-Gln-Pheol] and they are differing from each other in positions 6, 17 and 18, while Pept-1951-c and -d share the same general structure with an Aib/Vxx exchange in positions 9/10 (Table 1). Pept-1965-c-1, -c-2 and Pept-1966-d follow the same pattern as Pept-1951-c and -d with Ala and Lxx instead of Aib and Vxx in positions 9 and 10, respectively (Table 1). Regarding the Glu or Gln in position 18, acidic or neutral forms of these peptaibols were identified. Neutral longibrachin (LGB) A I-IV (18-Gln) [46] and acidic LGB II and III have been sequenced, and their antimicrobial activities against *Acholeplasma*, *Mycoplasma* and *Spiroplasma* cells as well as their strong membrane-permeabilizing action were described [26]. Trilongins BI–BIV and trilongin AI were also isolated from *T. longibrachiatum* by Mikkola et al. [48], who proved that there is a synergistic effect between the 11-residue and the 20-residue mitochondriotoxic trilongins of *T. longibrachiatum*. This opportunistically human pathogenic species has always been popular for its peptaibol production, which resulted in several studies naming the same compounds differently (longibrachins, trilongins or trichobrachins). Other species such as *T. aureoviride*, *T. koningii*, *T. polysporum*, *T. viride*, *T. orientale*, *T. phellinicola* and *Gliocladium deliquescens* can also produce peptaibols identical to compounds of *T. longibrachiatum*: trichoaureocins, trichokonins, trichosporins, suzukacillins, hyporientalins, hypophellins and gliodeliquescin A, respectively [49,53].

Since the differences in chemical structures and conformations of the peptaibols determine their variety of biological activities by the formation of pores in bilayer lipid membranes [9], we focused on the changes in the amounts of each peptaibol in the microheterogenous mixture to have a better insight into the correlation between the sequences of the peptaibols and their role in mycoparasitism. A previous work on the diversity profile of peptaibols produced by *T. aggressivum* and *T. pleuroti* in interactions with *A. bisporus* and *P. ostreatus* showed that the attacked host may also have an influence on the peptaibol profiles of *Trichoderma* [56]. In the recent study, monitoring the changes in the peptaibol profiles produced by *Trichoderma* species exposed to a pathogenic fungus in comparison with the unexposed control revealed a few differences in the amounts of some unique peptaibols, however the profiles of sequences within the particular microheterogenous mixtures remained similar in most of the cases. Tata et al. [69] monitored the secondary metabolites during the antagonistic interaction of *T. harzianum* and *Moniliophthora roreri* by desorption electrospray ionization mass spectrometry (DESI-MS) imaging, but the study did not focus on peptaibols, as it was assumed based on a previous work that peptaibol production is not pathogen-dependent [70]. In another study, matrix-assisted laser desorption/ionization mass spectrometry (MALDI-MS) has been established for the visualization of secondary metabolites in the mycoparasitic interaction between *T. atroviride* and *R. solani* by a thin layer of agar, and some signals related to peptaibols could be detected in the borders of fungal interaction [71]. However, this technique could not separately detect peptaibols with the same molecular weight but different sequences, which are usually present in the microheterogenous mixtures of these molecules.

Peptaibols and peptaibiotics, with their unusual amino acid content, are the products of non-ribosomal biosynthesis, and are assembled by the multienzyme thiotemplate mechanism [12,15]. Peptaibiotic NRPS genes are restricted to mycoparasitic lineages of Hypocreales. These genes are less conserved between species of *Tolypocladium*, but more conserved within the genus *Trichoderma* [72]. Since the comparison of the distribution of NRPS genes between saprophytic and parasitic species of *Trichoderma* supported the relevance of peptaibols in rhizosphere competition and biological control [51], the mRNA level of *tex1* was followed in *T. asperellum* both under control and confrontational conditions, which revealed an increasing expression during the interaction of *T. asperellum* with *R. solani*.

## 5. Conclusions

Several trichotoxins and longibrachins are produced by *T. asperellum* and *T. longibrachiatum*, respectively. The results of this study indicate that the total quantity of the detected peptaibol mixtures changes during in vitro confrontation of *Trichoderma* species with phytopathogenic fungi, which implicates the impact of the host on *Trichoderma* peptaibol metabolism. Such comparative studies will provide intriguing new insights into the physiology of *Trichoderma* species during mycoparasitism. It is conceivable that the amount of peptaibols may be involved in the regulation of biological control. The results provided a challenging opportunity to develop a deeper understanding of the underlying processes by which the amounts of peptaibol mixtures change during *Trichoderma*–plant pathogen interactions.

## Figures and Tables

**Table 1:** Peptaibol sequences produced by *T. asperellum* IRAN 3062C and *T. longibrachiatum* IRAN 3067C [46].

***T. asperellum* IRAN 3062C**	**Peak**	**M**	**b_12_**	**y_6_**	**R**	**R1**	**R2**	**R3**	**R4**	**R5**	**R6**	**R7**	**R8**	**R9**	**R10**	**R11**	**R12**	**R13**	**R14**	**R15**	**R16**	**R17**	**R18**		
Pept-1705-a-1	1	1705	1094	612	Ac	Aib	Gly	Aib	Lxx	Aib	Gln	Aib	Aib	Ala	Ser	Aib	Aib	Pro	Lxx	Aib	Aib	Gln	Vxxol		
Pept-1689-a-1	2	1689	1079	612	Ac	Aib	Gly	Aib	Lxx	Aib	Gln	Aib	Aib	Ala	Aib	Ala	Aib	Pro	Lxx	Aib	Aib	Gln	Vxxol		
Pept-1689-a-2	2	1689	1079	612	Ac	Aib	Gly	Aib	Lxx	Aib	Gln	Aib	Aib	Ala	Aib	Ala	Aib	Pro	Lxx	Aib	Aib	Gln	Vxxol		
Pept-1691-a-1	2	1691	1081	612	Ac	Aib	Gly	Aib	Lxx	Aib	Gln	Aib	Aib	Ala	Ser	Ala	Aib	Pro	Lxx	Aib	Aib	Gln	Vxxol		
Pept-1705-a-2	2	1705	1094	612	Ac	Aib	Gly	Aib	Lxx	Aib	Gln	Aib	Aib	Aib	Ser	Ala	Aib	Pro	Lxx	Aib	Aib	Gln	Vxxol		
Pept-1719-b-1	3	1719	1094	626	Ac	Aib	Gly	Aib	Lxx	Aib	Gln	Aib	Aib	Aib	Ser	Ala	Aib	Pro	Lxx	Aib	Vxx	Gln	Vxxol		
Pept-1719-a-1	3	1719	1108	612	Ac	Aib	Gly	Aib	Lxx	Aib	Gln	Aib	Aib	Aib	Ser	Aib	Aib	Pro	Lxx	Aib	Aib	Gln	Vxxol		
Pept-1705-a-3	3	1705	1094	612	Ac	Aib	Gly	Aib	Lxx	Aib	Gln	Aib	Aib	Aib	Ser	Ala	Aib	Pro	Lxx	Aib	Aib	Gln	Vxxol		
Pept-1703-a-1	3	1703	1092	612	Ac	Aib	Gly	Aib	Lxx	Aib	Gln	Aib	Aib	Aib	Aib	Ala	Aib	Pro	Lxx	Aib	Aib	Gln	Vxxol		
Pept-1749-b-1	3	1749	1123	626	Ac	Aib	Gly	Aib	Lxx	Aib	Gln	Aib	Aib	Aib	Ser	Vxx	Aib	Pro	Lxx	Aib	Vxx	Gln	Vxxol		
Trichotoxin T5D2/Pept-1675-a-1	4	1675	1064	612	Ac	Aib	Gly	Aib	Lxx	Aib	Gln	Aib	Aib	Ala	Ala	Ala	Aib	Pro	Lxx	Aib	Aib	Gln	Vxxol		
Pept-1719-b-2	4	1719	1094	626	Ac	Aib	Gly	Aib	Lxx	Aib	Gln	Aib	Aib	Aib	Ser	Ala	Aib	Pro	Lxx	Aib	Vxx	Gln	Vxxol		
Pept-1733-b-1	4	1733	1108	626	Ac	Aib	Gly	Aib	Lxx	Aib	Gln	Aib	Aib	Aib	Ser	Aib	Aib	Pro	Lxx	Aib	Vxx	Gln	Vxxol		
Trichotoxin A-50 F (T5F)/Pept-1689-b-1	4	1689	1064	626	Ac	Aib	Gly	Aib	Lxx	Aib	Gln	Aib	Aib	Ala	Ala	Ala	Aib	Pro	Lxx	Aib	Vxx	Gln	Vxxol		
Trichotoxin A-50 E (T5E)/Pept-1689-a-3	4	1689	1079	612	Ac	Aib	Gly	Aib	Lxx	Aib	Gln	Aib	Aib	Aib	Ala	Ala	Aib	Pro	Lxx	Aib	Aib	Gln	Vxxol		
Pept-1733-b-2	4	1733	1108	626	Ac	Aib	Gly	Aib	Lxx	Aib	Gln	Aib	Aib	Aib	Ser	Aib	Aib	Pro	Lxx	Aib	Vxx	Gln	Vxxol		
Trichotoxin A-50 F (T5F)/Pept-1689-b-2	5	1689	1064	626	Ac	Aib	Gly	Aib	Lxx	Aib	Gln	Aib	Aib	Ala	Ala	Ala	Aib	Pro	Lxx	Aib	Vxx	Gln	Vxxol		
Pept-1733-b-3	5	1733	1108	626	Ac	Aib	Gly	Aib	Lxx	Aib	Gln	Aib	Aib	Aib	Ser	Aib	Aib	Pro	Lxx	Aib	Vxx	Gln	Vxxol		
Trichotoxin sequence 05/Pept-1703-a-2	5	1703	1092	612	Ac	Aib	Gly	Aib	Lxx	Aib	Gln	Aib	Aib	Aib	Ala	Aib	Aib	Pro	Lxx	Aib	Aib	Gln	Vxxol		
Pept-1703-b-1	5	1703	1078	626	Ac	Aib	Gly	Aib	Lxx	Aib	Gln	Aib	Ala	Aib	Ala	Aib	Aib	Pro	Lxx	Aib	Vxx	Gln	Vxxol		
Pept-1733-b-4	5	1733	1108	626	Ac	Aib	Gly	Aib	Lxx	Aib	Gln	Aib	Aib	Aib	Ser	Aib	Aib	Pro	Lxx	Aib	Vxx	Gln	Vxxol		
Pept-1703-b-2	6	1703	1078	626	Ac	Aib	Gly	Aib	Gln	Aib	Gln	Aib	Aib	Aib	Gly	Ala	Aib	Pro	Lxx	Aib	Vxx	Gln	Vxxol		
Pept-1747-b-1	6	1747	1122	626	Ac	Aib	Gly	Aib	Gln	Aib	Gln	Aib	Aib	Aib	Aib	Aib	Aib	Pro	Lxx	Aib	Vxx	Gln	Vxxol		
Pept-1703-b-3	6	1703	1078	626	Ac	Aib	Gly	Aib	Lxx	Aib	Gln	Aib	Ala	Aib	Ala	Aib	Aib	Pro	Lxx	Aib	Vxx	Gln	Vxxol		
Pept-1733-b-5	6	1733	1108	626	Ac	Aib	Gly	Aib	Lxx	Aib	Gln	Aib	Aib	Aib	Ser	Aib	Aib	Pro	Lxx	Aib	Vxx	Gln	Vxxol		
Trichotoxin A-50 I (T5I)/Pept-1717-b-1	6	1717	1092	626	Ac	Aib	Gly	Aib	Lxx	Aib	Gln	Aib	Aib	Aib	Ala	Aib	Aib	Pro	Lxx	Aib	Vxx	Gln	Vxxol		
Pept-1731-b-1	7	1731	1106	626	Ac	Aib	Ala	Aib	Lxx	Aib	Gln	Aib	Aib	Aib	Ala	Aib	Aib	Pro	Lxx	Aib	Vxx	Gln	Vxxol		
Trichotoxin A-50J (T5J)/Pept-1731-b-2	8	1731	1106	626	Ac	Aib	Gly	Aib	Lxx	Aib	Gln	Aib	Aib	Aib	Aib	Aib	Aib	Pro	Lxx	Aib	Vxx	Gln	Vxxol		
General structure		-	-	-	Ac	Aib	◆	Aib	●	Aib	Gln	Aib	■	■	🟄	⬟	Aib	Pro	Lxx	Aib	▲	Gln	Vxxol		

***T. longibrachiatum* IRAN 3067C**	**Peak**	**M**	**b_13_**	**y_7_**	**R**	**R1**	**R2**	**R3**	**R4**	**R5**	**R6**	**R7**	**R8**	**R9**	**R10**	**R11**	**R12**	**R13**	**R14**	**R15**	**R16**	**R17**	**R18**	**R19**	**R20**
Longibrachin A II/Trilongin B II/Pept-1951-d	1	1951	1974	789	Ac	Aib	Ala	Aib	Ala	Aib	Ala	Gln	Aib	Vxx	Aib	Gly	Lxx	Aib	Pro	Vxx	Aib	Vxx	Glu	Gln	Pheol
Longibrachin A I/Trilongin B I/Pept-1936-a	1	1936	1959	774	Ac	Aib	Ala	Aib	Ala	Aib	Ala	Gln	Aib	Vxx	Aib	Gly	Lxx	Aib	Pro	Vxx	Aib	Aib	Gln	Gln	Pheol
Longibrachin B II/Trilongin C I/Pept-1938-b	1	1938	1961	775	Ac	Aib	Ala	Aib	Ala	Aib	Ala	Gln	Aib	Vxx	Aib	Gly	Lxx	Aib	Pro	Vxx	Aib	Aib	Glu	Gln	Pheol
Pept-1951-c	1	1951	1974	788	Ac	Aib	Ala	Aib	Ala	Aib	Ala	Gln	Aib	Aib	Vxx	Gly	Lxx	Aib	Pro	Vxx	Aib	Vxx	Gln	Gln	Pheol
Pept-1952-d	1	1952	1975	789	Ac	Aib	Ala	Aib	Ala	Aib	Ala	Gln	Aib	Aib	Vxx	Gly	Lxx	Aib	Pro	Vxx	Aib	Vxx	Glu	Gln	Pheol
Longibrachin A III/Pept-1951-a	2	1951	1974	774	Ac	Aib	Ala	Aib	Ala	Aib	Aib	Gln	Aib	Vxx	Aib	Gly	Lxx	Aib	Pro	Vxx	Aib	Aib	Gln	Gln	Pheol
Longibrachin B III/Trilongin C III/Pept-1951-b	2	1951	1974	775	Ac	Aib	Ala	Aib	Ala	Aib	Aib	Gln	Aib	Vxx	Aib	Gly	Lxx	Aib	Pro	Vxx	Aib	Aib	Glu	Gln	Pheol
Pept-1965-c-1	3	1965	1988	788	Ac	Aib	Ala	Aib	Ala	Aib	Aib	Gln	Aib	Ala	Lxx	Gly	Lxx	Aib	Pro	Vxx	Aib	Vxx	Gln	Gln	Pheol
Pept-1966-d	3	1966	1988	789	Ac	Aib	Ala	Aib	Ala	Aib	Aib	Gln	Aib	Ala	Lxx	Gly	Lxx	Aib	Pro	Vxx	Aib	Vxx	Glu	Gln	Pheol
Pept-1965-c-2	4	1965	1988	788	Ac	Aib	Ala	Aib	Ala	Aib	Aib	Gln	Aib	Ala	Lxx	Gly	Lxx	Aib	Pro	Vxx	Aib	Vxx	Gln	Gln	Pheol
General structure		-	-	-	Ac	Aib	Ala	Aib	Ala	Aib	■	Gln	Aib	⬟	🟎	Gly	Lxx	Aib	Pro	Vxx	Aib	▲	▼	Gln	Pheol

●: Lxx/Gln; ■: Aib/Ala; ▲: Vxx/Aib; ▼: Gln/Glu; ◆: Vxxol/Lxxol; ⬟: Aib/Ala/Vxx; 🟎: Aib/Vxx/Lxx; 🟄: Ser/Aib/Ala/Gly.
